# Identification of hub genes and transcription factors in patients with rheumatoid arthritis complicated with atherosclerosis

**DOI:** 10.1038/s41598-022-08274-1

**Published:** 2022-03-18

**Authors:** Lu Xiao, Zhou Yang, Shudian Lin

**Affiliations:** Department of Rheumatology, Hainan General Hospital (Hainan Affiliated Hospital of Hainan Medical University), Hainan, 570311 China

**Keywords:** Rheumatic diseases, Cardiovascular diseases

## Abstract

The aim of this study was to explore the overlapping key genes, pathway networks and transcription factors (TFs) related to the pathogenesis of rheumatoid arthritis (RA) and atherosclerosis. The gene expression profiles of RA and atherosclerosis were downloaded from the Gene Expression Omnibus database. Differentially expressed genes (DEGs) between RA and atherosclerosis were identified. The biological roles of common DEGs were explored through enrichment analysis. Hub genes were identified using protein–protein interaction networks. TFs were predicted using Transcriptional Regulatory Relationships Unraveled by Sentence Based Text Mining (TRRUST) database. The hub genes and TFs were validated with other datasets. The networks between TFs and hub genes were constructed by CytoScape software. A total of 131 DEGs (all upregulated) were identified. Functional enrichment analyses indicated that DEGs were mostly enriched in leukocyte migration, neutrophil activation, and phagocytosis. CytoScape demonstrated 12 hub genes and one gene cluster module. Four of the 12 hub genes (CSF1R, CD86, PTPRC, and CD53) were validated by other datasets. TRRUST predicted two TFs, including Spi-1 proto-oncogene (SPI1) and RUNX family transcription factor 1(RUNX1). The expression of RUNX1 was validated with another dataset. Our study explored the common pathogenesis of RA and atherosclerosis. These results may guide future experimental research and clinical transformation.

## Introduction

Rheumatoid arthritis (RA), a chronic autoimmune disease of unknown etiology, is characterized by synovitis, pannus formation, and destruction of joints and cartilage. RA-related health complications and long-term comorbidities can markedly decrease life expectancy, and cardiovascular morbidity and mortality are relatively high. Cardiovascular diseases and sudden death are the main reasons of premature mortality in patients with RA^1^. Approximately 30%–60% increase in risk of cardiovascular events and 50% increase in risk of death due to cardiovascular disease were reported in patients with RA because of accelerated atherosclerosis^2^.

Evidence supports the hypothesis that the pathology of RA and that of atherosclerosis pathology share many common molecular pathways and exhibit analogous mechanisms^3^. Atherosclerotic plaque has many similarities to RA synovium, such as recruiting of blood-borne mononuclear cells, upregulation of cytokines, and complex interactions of immune cells with resident cell types^3^. The common pathophysiological mechanisms may provide insights into the common pathogenesis of RA and atherosclerosis. Therefore, the relationship between these diseases has attracted increasingly interest. Tamami et al. reported RA patients who have visceral adiposity have a specifically high risk for atherosclerosis^4^. In 2020, a study proposed the use of QRESEARCH risk estimator version 3 and the European League Against Rheumatism mSCORE for identifying patients with RA who are at a high risk of carotid plaques^5^. In addition, Dessein et al. determined the performance of the Framingham score and the Systematic Coronary Risk Evaluation tool in assessing patients with RA who are at high risk of atherosclerosis^6^. Furthermore, the development of atherosclerosis in patients with RA seems to be influenced by a genetic component. This topic has been the focus of numerous bioinformatics studies that mostly concentrated on genetic polymorphisms. To be specific, the association of the paraoxonase 1 gene polymorphism with carotid plaques in RA was demonstrated in a series of 168 North American patients ^7^. Moreover, a Spanish group proved the potential protective effect of the IL33 rs3939286 allele T against the risk of subclinical atherosclerosis in patients with RA. However, a number of studies have also reported negative results on this aspect. One study failed to confirm the association of CRP, HNF1A, LEPR, GCKR, NLRP3, IL1F10, PPP1R3B, ASCL1, HNF4A, and SALL1 with cardiovascular disease in RA^8^. Another work reported that 15 polymorphisms, including TCF21, LPA, HHIPL1, RASD1-PEMT, MRPS6, CYP17A1-CNNM2-NT5C2, SMG6-SRR, PHACTR1, WDR12, and COL4A1-COL4A2, are not associated with atherosclerosis in RA^9^. Meanwhile, no relationships among ABO rs579459, PPAP2B rs17114036, ADAMTS7 rs3825807, PIK3CG rs17398575, EDNRA rs1878406, and subclinical atherosclerosis in RA were found^10^. Therefore, in consideration of previous studies, the molecular mechanism in patients with RA complicated with atherosclerosis is controversial and needs further study.

With the rapid development of microarray techniques, differentially expressed genes (DEGs) among different groups of people can be detected. Microarray techniques can illustrate gene expression and identify special proteins produced by genes^11^. Therefore, disease-related molecules can be identified by using these tools. Thus, in this study, we aim to explore the potential overlapping key genes, pathway networks, and transcription factors (TFs) related to the pathogenesis of RA and atherosclerosis through a combination of microarray and bioinformatics analyses.

## Results

### Identification of common DEGs

DEGs were identified after the microarray results were standardized (Supplementary Fig. 1a–d). A total of 2735 DEGs were found in the RA datasets (GSE55235 and GSE55457), and 275 DEGs were found in the atherosclerosis dataset (GSE14905). Supplementary Fig. 1a shows the volcano plot of GSE55235 and GSE55457 (RA). Supplementary Fig. 1b provides the volcano plot of GSE14905 (atherosclerosis). The heatmap of the RA datasets (GSE55235 and GSE55457) is presented in Supplementary Fig. 1c, and the heatmap of the atherosclerosis dataset (GSE14905) is depicted in Supplementary Fig. 1d. A total of 131 common DEGs were found after the integration of the DEGs (Supplementary Fig. 1e).

### Protein–protein interaction (PPI) network construction, biological functions analyses, and Molecular Complex Detection (MCODE) cluster modules identification

**Figure 1:**
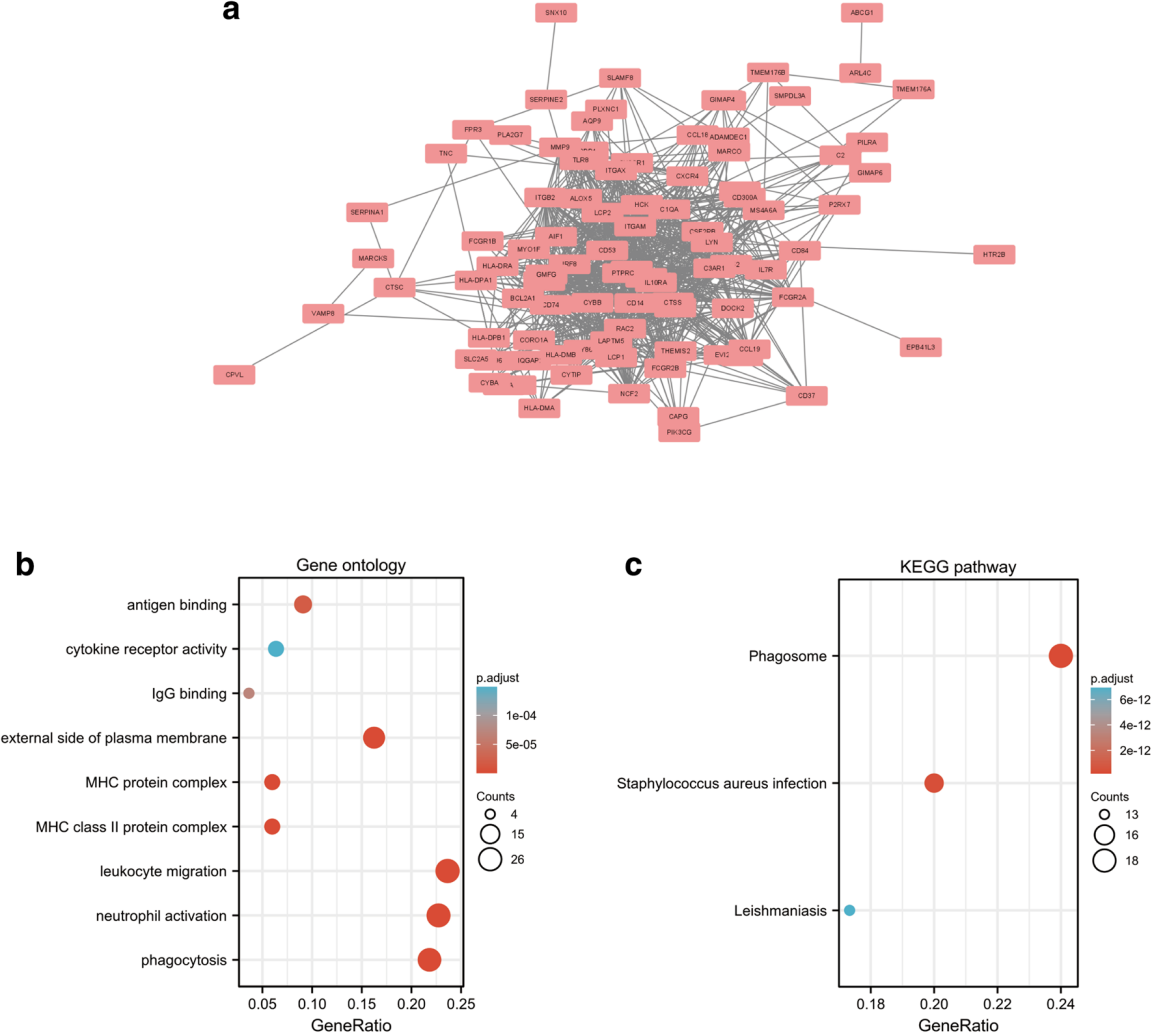
PPI network and functional enrichment of DEGs. (**a**) The interaction network between proteins coded by DEGs. (**b**) The enrichment analysis results of GO and (**c**) KEGG (www.kegg.jp/kegg/kegg1.html) pathway. Adjusted P value < 0.05 was considered significant.

The PPI network for the 131 DEGs was constructed after the common DEGs were imported to STRING (Fig. 1a). Gene Ontology (GO) and Kyoto Encyclopedia of Genes and Genomes (KEGG) analyses were used in analyzing the 131 common DEGs (Fig. 1b,c)^12–14^. Based on GO enrichment, the biological process acted primarily on phagocytosis, leukocyte migration, and neutrophil activation. These proteins were primarily located in external side of plasma membrane, MHC class II protein complex, and MHC protein complex. With regard to molecular functions, the proteins played roles in antigen binding, IgG binding, and cytokine receptor activity (Fig. 1b). According to KEGG pathway analysis, these proteins were primarily involved in phagosome, staphylococcus aureus infection, and leishmaniasis (Fig. 1c).

**Figure 2:**
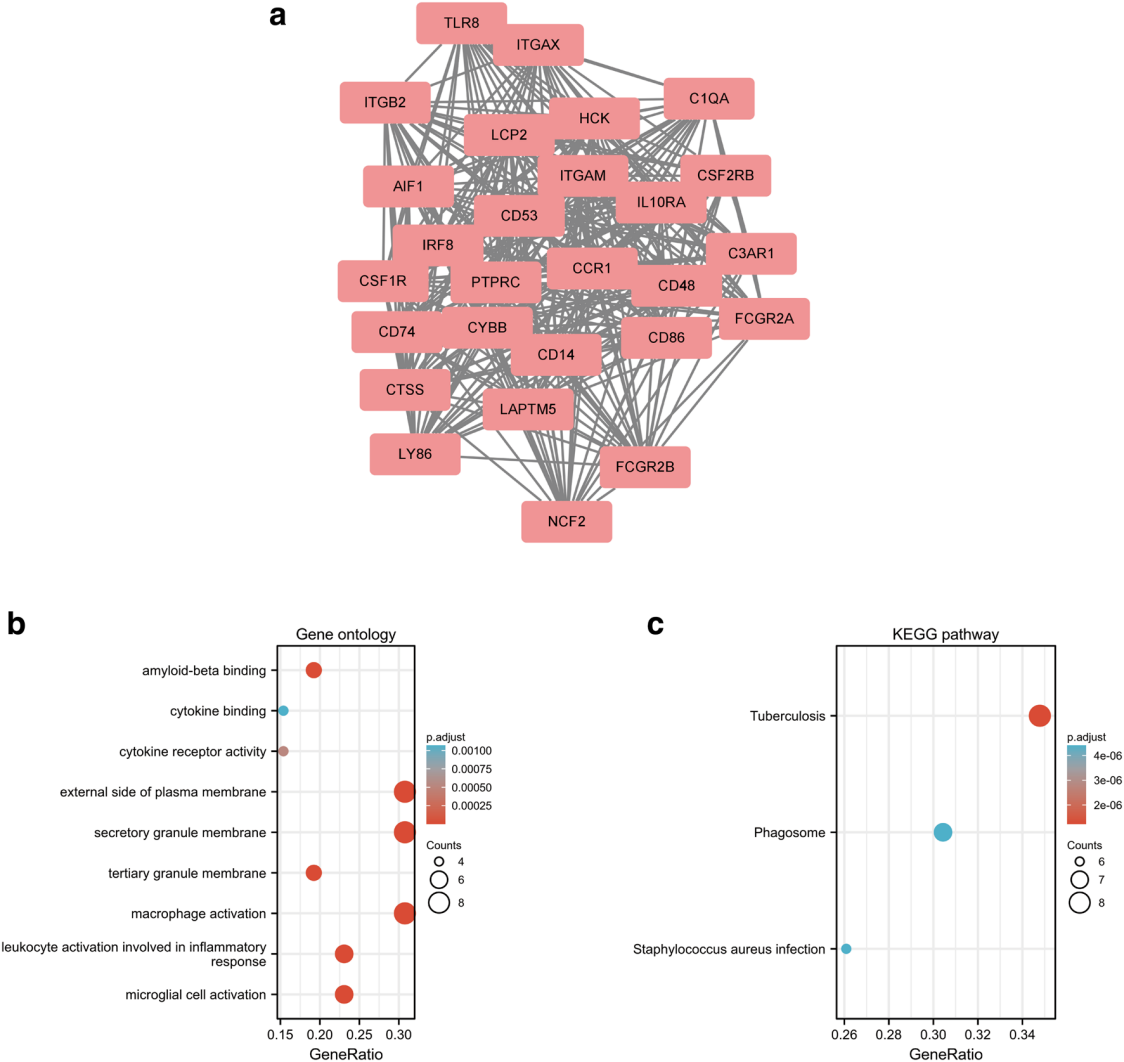
Cluster modules extracted by MCODE and enrichment analysis of the modular genes. (**a**) One significant gene clustering module. (**b**) GO and (**c**) KEGG enrichment analysis of the modular genes. Adjusted P value < 0.05 was considered significant.

Significant modules of the PPI network were identified by MCODE. MCODE score of 4 was set as a threshold. One module with MCODE score of ≥ 4 is illustrated in Fig. 2a. This cluster (MCODE score = 24.385) had 27 nodes and 317 edges (Fig. 2a). GO analysis showed that the proteins in the cluster were related to macrophage activation, microglial cell activation, and leukocyte activation (Fig. 2b). KEGG pathway analysis showed that these proteins were primarily involved in tuberculosis, phagosome, and staphylococcus aureus infection (Fig. 2c).

### Selection and analysis of hub genes

**Figure 3:**
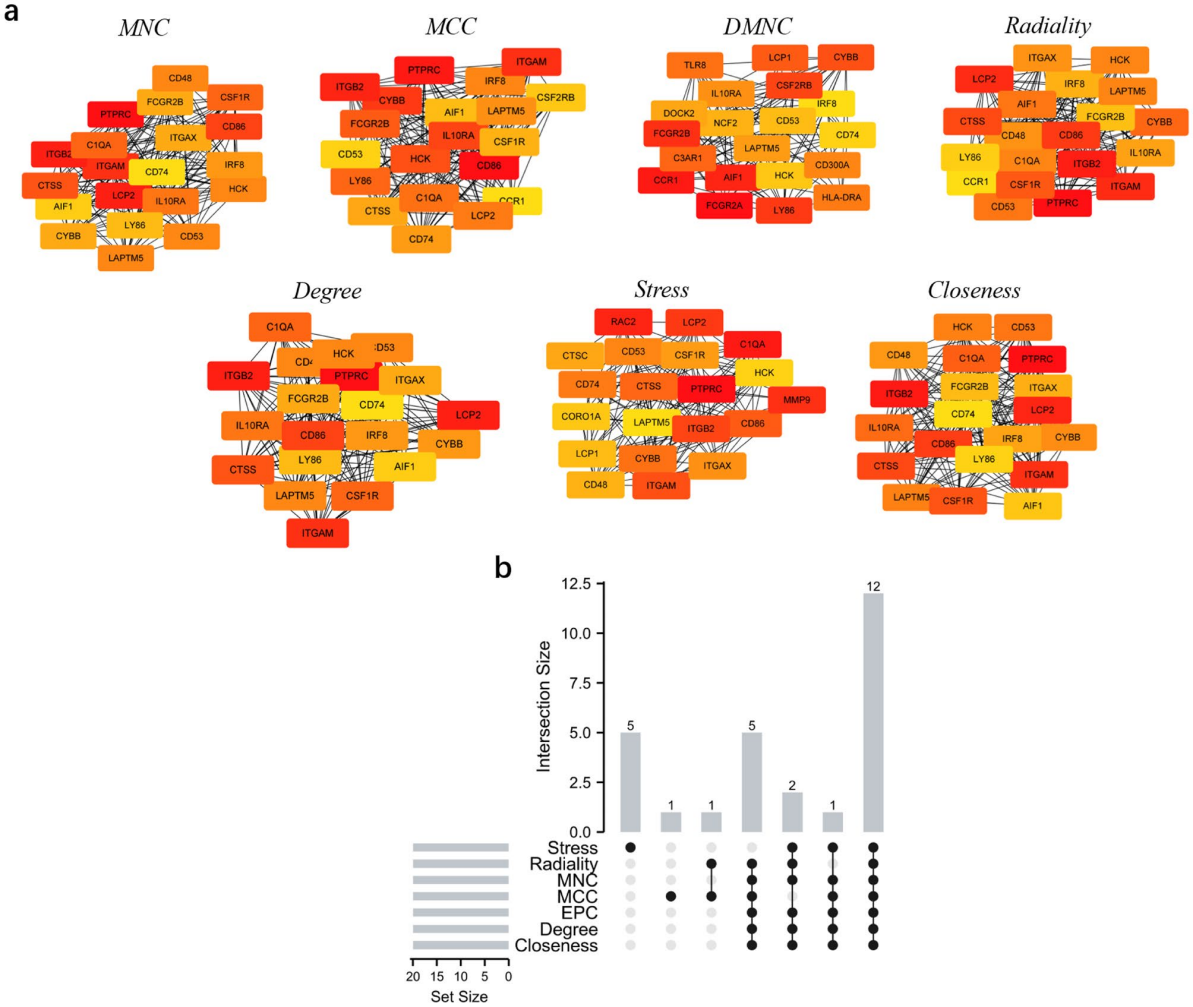
Hub genes identified by different algorithms and UpSet diagram. (**a**) Hub gene identified by seven different algorithms. (**b**) The UpSet diagram showed that the seven algorithms screened 12 overlapping hub genes.

**Figure 4:**
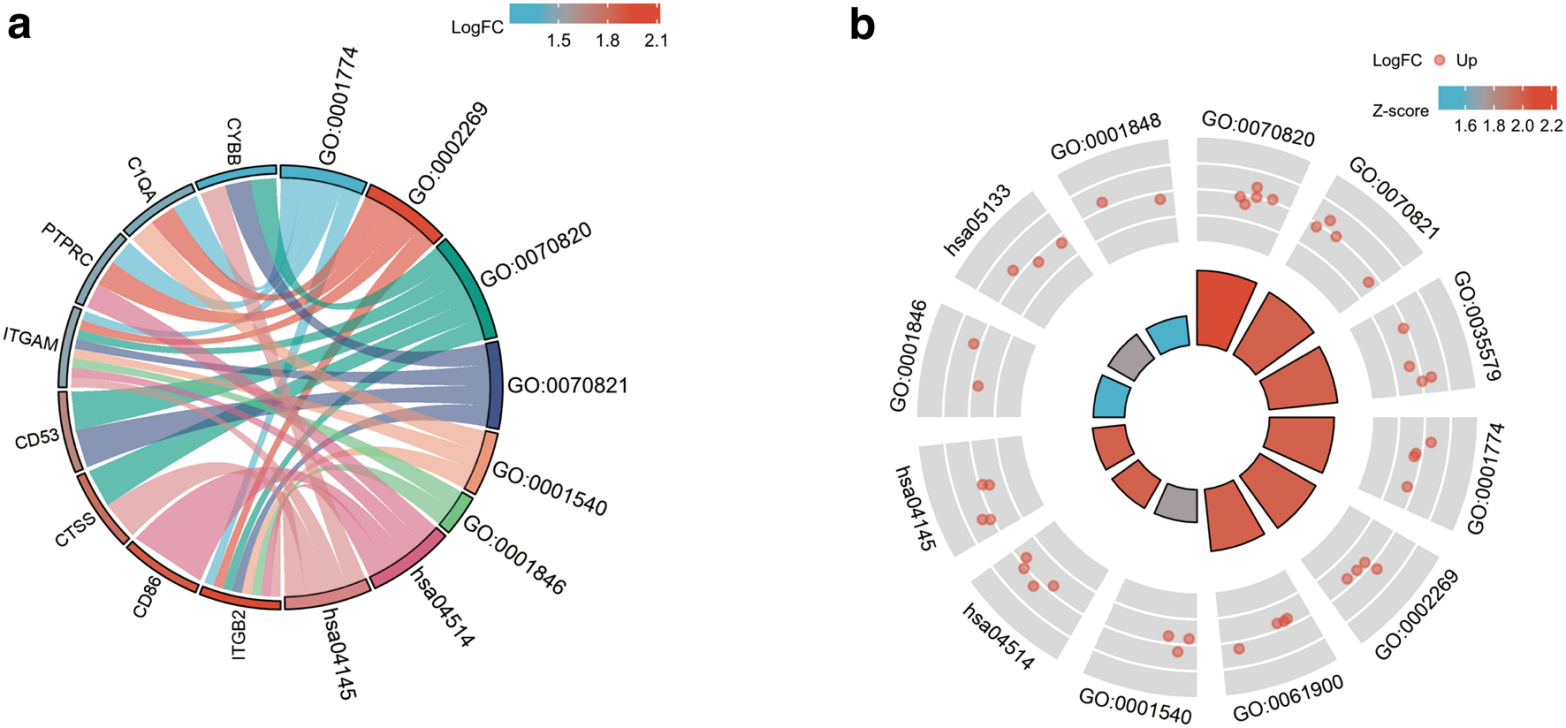
GO and KEGG enrichment analysis of the hub genes.

The top 20 hub genes were calculated using the seven algorithms of the plug-in CytoHubba (Fig. 3a). After the intersection of the UpSet diagram was determined, 12 common hub genes were discovered, namely, cytochrome b-245 beta chain (CYBB), lysosomal protein transmembrane 5 (LAPTM5), colony stimulating factor 1 receptor (CSF1R), Src family tyrosine kinase (HCK), integrin subunit alpha M (ITGAM), CD86 molecule (CD86), complement C1q A chain (C1QA), integrin subunit beta 2 (ITGB2), protein tyrosine phosphatase receptor type C (PTPRC), cathepsin S (CTSS), lymphocyte cytosolic protein 2 (LCP2), and CD53 molecule (CD53; Fig. 3b). Table 1 shows their full names and related functions. GO analysis showed that the genes were mainly involved in amyloid-beta binding, opsonin binding, and complement binding. KEGG pathway analysis revealed that the hub genes were primarily involved in cell adhesion molecules, phagosome, and pertussis (Fig.4a,b).

**Table 1:** The details of the hub genes.

Gene symbol	Full name	Function
CYBB	cytochrome b-245 beta chain	CYBB is an important composition of cytochrome b (−245), which has been proposed as a primary component of the microbicidal oxidase system of phagocytes^45^
LAPTM5	lysosomal protein transmembrane 5	LAPTM5, encoding a lysosome-associated protein, E3 protein. This protein may play a role in hematopoiesis^46^
CSF1R	colony stimulating factor 1 receptor	CSF1R encoded protein is the receptor for colony stimulating factor 1, which is a kind of cytokines controls the production, differentiation, and function of macrophages^21^
HCK	Src family tyrosine kinase	The protein encoded by HCK is a member of the Src family of tyrosine kinases, which play a role in neutrophil migration and in the degranulation of neutrophils^47^
ITGAM	integrin subunit alpha M	This gene encodes the integrin alpha M chain, which is important in the adherence of neutrophils and monocytes to stimulated endothelium, and also in the phagocytosis of complement coated particles^48^
CD86	CD86 molecule	CD86 encodes a type I membrane protein. CD28 antigen binds to this protein to generate a costimulatory signal for activation of the T-cell. Cytotoxic T-lymphocyte-associated protein 4 binds to this protein to negatively regulate T-cell activation and diminishes the immune response^49^
C1QA	complement C1q A chain	C1QA encodes C1r and C1s associated proteins to yield the first component of the serum complement system. The deficiency is related to lupus erythematosus and glomerulonephritis^50^
ITGB2	integrin subunit beta 2	This gene encodes an integrin beta chain. The encoded protein mainly affects immune response. The deficiency of ITGB2 cause leukocyte adhesion deficiency^51^
PTPRC	protein tyrosine phosphatase receptor type C	The protein encoded by this gene is a member of the protein tyrosine phosphatase family. The family is known to be signaling molecules that regulates a variety of cellular processes including cell growth, differentiation, mitosis, and oncogenic transformation^52^
CTSS	cathepsin S	The preproprotein encoded by this gene is a lysosomal cysteine proteinase that participates in the degradation of antigenic proteins to peptides for presentation on MHC class II molecules^53^
LCP2	lymphocyte cytosolic protein 2	This gene encodes an adapter protein,which is thought to play a role in TCR-mediated intracellular signal transduction^54^
CD53	CD53 molecule	The encoded protein contributes to the transduction of CD2-generated signals in T cells and natural killer cells and has been suggested to play a role in growth regulation^55^

### Validation of hub genes expression

**Figure 5:**
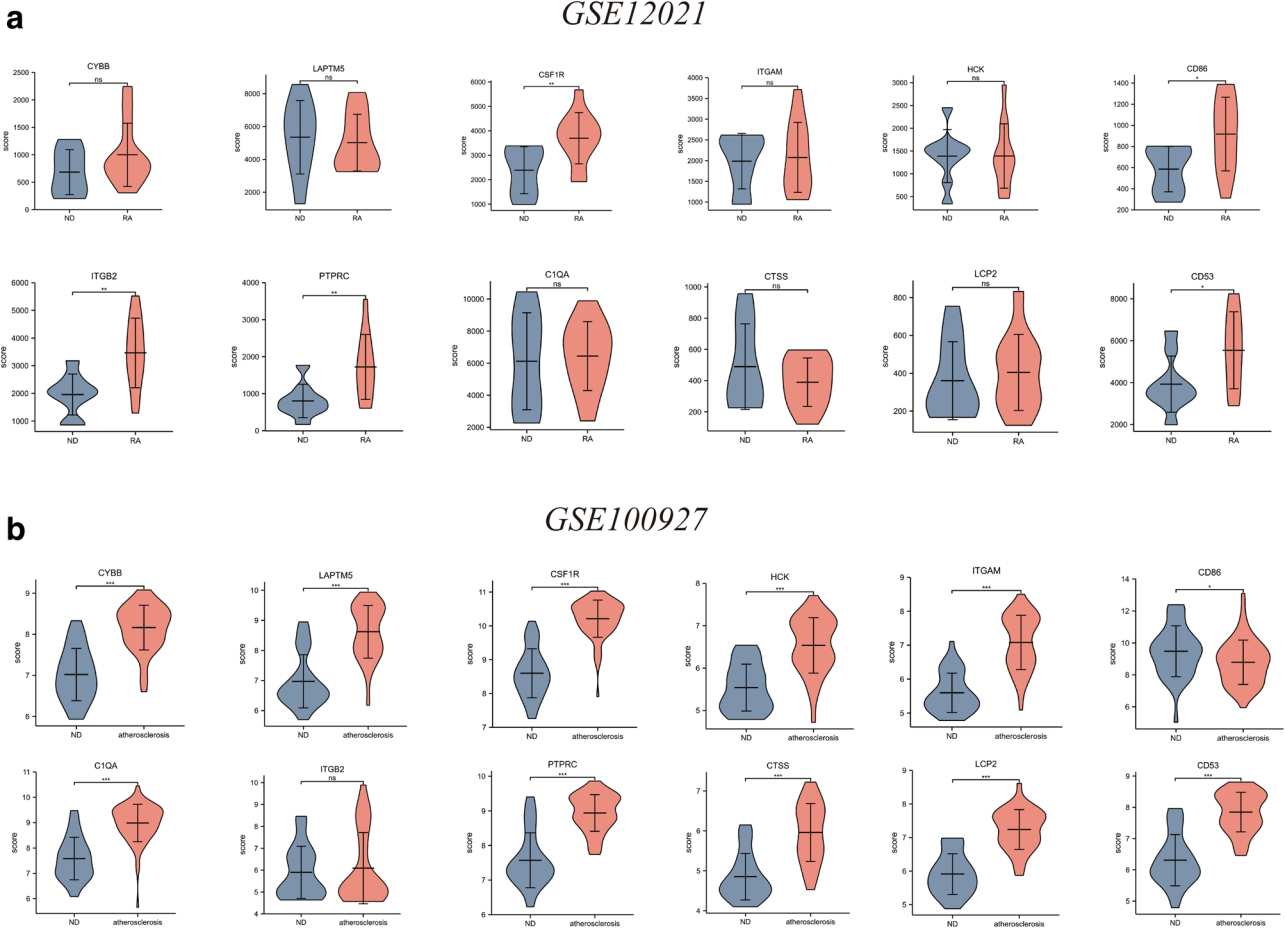
Expression level of hub gene in GSE12021 and GSE100927. (**a**) The verification of hub genes in GSE12021. (**b**) The verification of hub genes in GSE100927 The comparison between the two sets of data with the mean T test. RStudio (https://www.R-project.org) was used in statistical analysis. P value < 0.05 was considered statistically significant. *P < 0.05; ***P < 0.001.

The GSE12021 and GSE100927 datasets were used in verifying the expression of the identified hub genes. The mRNA expression levels of CSF1R, CD86, ITGB2, PTPRC, and CD53 were significantly increased in the RA samples compared with the ND samples (P < 0.05, Fig. 5a). In addition, the mRNA expression levels of CYBB, LAPTM5, CSF1R, HCK, ITGAM, CD86, C1QA, PTPRC, CTSS, LCP2, and CD53 were significantly increased in the atherosclerotic plaque samples (P < 0.05, Fig. 5b).

### Prediction and verification of TFs

**Figure 6:**
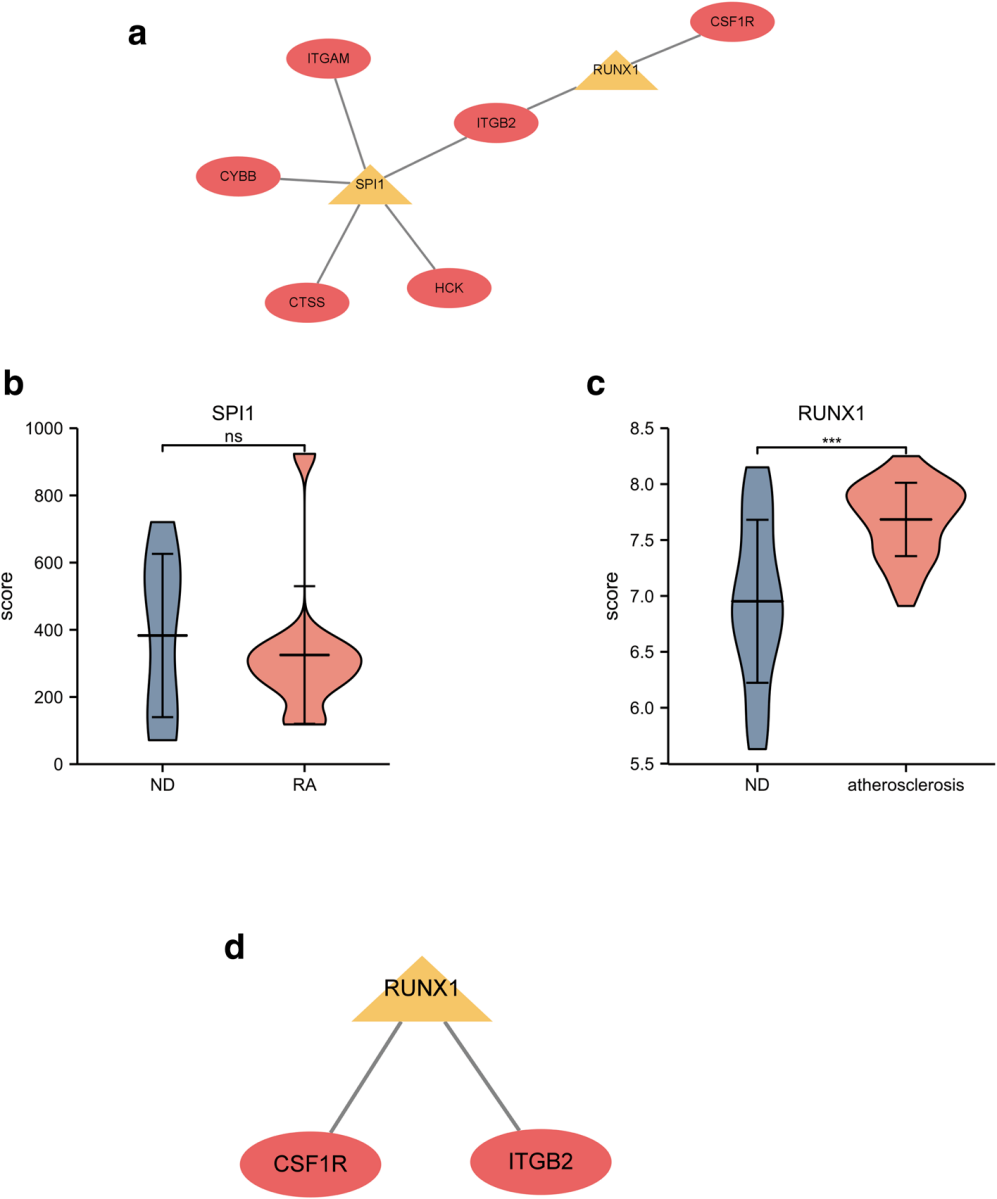
TFs regulatory network and its expression in GSE12021. (**a**) TFs regulatory network. (**b,c**) The expression level of TFs in GSE12021. *P < 0.05. (**d**) TF regulatory network that has been verified. TFs were marked in yellow, and the hub genes were marked in red.

Two TFs that may regulate the expression of the hub genes were identified on the basis of the Transcriptional Regulatory Relationships Unraveled by Sentence Based Text Mining (TRRUST) database (Fig. 6a and Table 2). Spi-1 proto-oncogene (SPI1) and RUNX family transcription factor 1 (RUNX1) were predicted to have the capability to regulate six hub genes (including ITGAM, CYBB, CTSS, HCK, ITGB2, and CSF1R) by acting as TFs. During further verification, we found that one of the TFs, RUNX1, had low expression in the synovial samples of patients with RA in the GSE12021 dataset, whereas the expression of SPI1 was not significantly changed (P < 0.05, Fig. 6b,c). RUNX1, which may function as an important TF in the pathogenesis of patients with RA complicated with atherosclerosis, was predicted to participate in the regulation of the two hub genes CSF1R and ITGB2 (Fig. 6d).

**Table 2:** Key transcriptional factors (TFs) of hub genes.

Key TFs	Description	P-value	genes
SPI1	spleen focus forming virus (SFFV) proviral integration oncogene spi1	2.52E-10	CTSS, CYBB, HCK, ITGAM, ITGB2
RUNX1	runt-related transcription factor 1	0.000285	CSF1R, ITGB2

## Discussion

The main purpose of our study is to identify the common DEGs in RA and atherosclerosis for the prediction of RA complicated with atherosclerosis and the revelation of potential mechanisms. In this study, 131 overlapping DEGs (all upregulated), of which 12 were hub genes, were detected in both diseases. Among those of the 12 hub genes, the expression levels of CSF1R, CD86, PTPRC, and CD53 were verified by using synovial and atherosclerotic plaque samples. GO and KEGG pathway enrichment analyses revealed that the genes were significantly enriched in phagocytosis, leukocyte migration, and neutrophil activation. In addition, two TFs, RUNX1 and SPI1, were predicted to play roles in the pathogenesis process. One of the TFs, RUNX1, was verified to have low expression in the synovial samples of patients with RA.

Our results from GO and KEGG pathway enrichment analyses indicated that phagocytosis seemed to be activated in RA complicated with atherosclerosis. Prior studies have revealed that the key role of macrophages in atherosclerosis is the phagocytosis of apoptotic and necrotic cells and cell debris; such a role may help reverse plaque vulnerability^15^. Phagocytosis is impaired in human atherosclerotic plaques, and CD47-blocking antibodies can restore phagocytosis to prevent atherosclerosis^16^. However, in our study, phagocytosis appeared to be activated. This result may indicate that the mechanism of atherosclerosis complicated with autoimmune diseases, such as RA, may be different. In the disease state, chronic inflammation can lead to the aberrant remodeling of macrophage responses and cause a shift in macrophage phenotypes^17^. In RA, proinflammatory macrophages are extremely upregulated, which may induce the activation of phagocytosis. Given that this process is always highly activated, phagocytosis may not be a protective factor in patients with RA complicated with atherosclerosis. The exact role of phagocytosis is an interesting study direction and may need further investigation. In addition, our study showed that leukocyte migration and neutrophil activation were also vital in the pathogenesis process. The vasculature is important in inflammation, angiogenesis, and atherosclerosis associated with the pathogenesis of RA^18^. Vascular injury is caused primarily by activated neutrophils and inflammatory mediators released by neutrophils^19^. In RA, leukocytes can migrate into the synovium and cause subsequent inflammation processes^20^. Therefore, the inhibition of phagocytosis, leukocyte migration, and neutrophil activation may offer potential treatment options for patients with RA complicated with atherosclerosis.

Furthermore, our study identified hub genes by using seven common algorithms. Among those of the 12 identified hub genes, the expression levels of CSF1R, CD86, PTPRC, and CD53 were verified. CSF1 is a cytokine that controls the production, differentiation, and function of macrophages^21^. Although CSF1R is a target in RA and atherosclerosis treatment, studies directly analyzing its role in the concomitant two diseases are limited. Our study demonstrated that CSF1R had remarkably increased expression and served as one of the hub genes. Therefore, drugs targeting CSF1R may have great potential in controlling both diseases simultaneously. CD86 is a type I membrane protein. The CD28 antigen binds to CD86 and generates a co-stimulatory signal for T-cell activation. Abatacept, one of the treatment drugs for RA, can modulate the CD80/CD86–CD28 co-stimulatory signal required for T-cell activation. Although many studies have reported improvement in lipid profiles after abatacept treatment, most of these studies attributed this effect to the reduction in general inflammation and disease activity^22,23^. Our study showed that CD86 played a vital role in the pathogenesis of RA with atherosclerosis. This role may be another reason for the improvement in lipid profiles after abatacept treatment. PTPRC, also known as CD45, regulates a variety of cellular processes^24^. Patients with RA who harbor a particular mutation at the PTPRC gene s10919563 locus have better responses to antitumor necrosis factor therapy than individuals who lack it^25,26^. Meanwhile, PTPRC acts as a regulatory T cell (Treg)-related gene in the deterioration of atherosclerosis^27^. The upregulation of PTPRC is associated with the progression of atherosclerosis^27^. Therefore, considering its role in RA and atherosclerosis, PTPRC is a possible treatment target. CD53 regulates the growth of T cells and natural killer cells and has been extensively studied in RA^28^. CD53 was elevated significantly on leukocytes from patients with RA compared with leukocytes from controls^29^. Meanwhile, CD53 curbs inflammatory cytokine secretion and is regarded as immune-responsive hub genes related to advanced plaques^30^. Therefore,drugs targeting CD53 are able to control both dieases at the same time, which emphasizes its importance in future research.

Our study also predicted the TFs of identified hub genes. RUNX1, which was significantly downregulated in RA synovium samples, regulated two hub genes, namely, CSF1R and ITGB2. A previous study discovered that the enhancer of zeste homolog 2 in CD4+ T cells from patients with RA was attenuated, downregulated RUNX1, and ultimately suppressed Treg differentiation^31^. Treg plays a pivotal role in maintaining immune homeostasis, and Treg deficiency is implicated in the pathogenesis of RA^32,33^. In addition, Treg is highly involved in atherogenesis. Treg plays a key atheroprotective role by limiting inflammation and counterbalancing plaque formation^34^.Therefore, the downregulated of RUNX1 may cause Treg suppression, which may aggravate the atherogenesis in RA patinets. Meanwhile, SPI1 was also predicted to be a vital TF in patients with RA complicated with atherosclerosis. However, its expression did not change dramatically likely because of the small smple size of GSE12021. The expression of the SPI1 gene is upregulated during the differentiation of myeloid cells^35^. In 2020, Pan et al. demonstrated that the expression of SPI1 is significantly increased after a high-fat/cholesterol diet; this result implied that the activation of myeloid cells is critical for atherosclerosis development^36^. A previous study proved that the inhibition of the complex formed by SPI1 led to the reduction in atherosclerotic lesions^37,38^. Our study predicted that SPI1 can regulate five hub genes. However, GSE12021 failed to verify the expression level of the five hub genes and SPI1. Hence, the role of SPI1 may need further assessment in a study with a precise design and large population.

In conclusion, our study aimed to identify and verify possible hub genes and TFs, which may serve as promising treatment targets for patients with RA complicated with atherosclerosis. CSF1R, CD86, PTPRC, and CD53 were identified and verified as hub genes. GO and KEGG pathway enrichment analyses revealed that these genes were significantly enriched in phagocytosis, leukocyte migration, and neutrophil activation. In addition, RUNX1 was predicted to participates in the regulation of hub genes as a TF, indicating the role of Treg in this process.

## Materials and methods

### Data collection

‘‘Rheumatoid arthritis’’ or “atherosclerosis” were used as key words for the expression profiling of RA or atherosclerosis in the GEO database, which is a public repository database^39^. Datasets, including synovial biopsies from RA or plaque biopsies from atherosclerosis, were used. Finally, three datasets, namely, GSE55235 (GPL96), GSE55457 (GPL96), and GSE28829 (GPL570), were selected as test sets. GSE55235 (GPL96) and GSE55457 (GPL96) included synovial biopsies from 23 patients with RA and 20 normal donors (NDs). GSE28829(GPL570) included plaque biopsies from 13 intimal thickening and 16 thick fibrous cap atheroma lesions. Two datasets, namely, GSE12021 (GPL96) and GSE100927 (GPL17077), were selected as validation sets for RA and atherosclerosis, respectively. The overall flowchart of this research is shown in Supplementary Fig. 2.

### Identification of DEGs

The row expression data of GSE55235, GSE55457, and GSE28829 were analyzed. DEGs between the disease and ND groups were obtained using the online web-based tool GEO2R. |Log2 fold change|> 1 and adjusted P value < 0.05 were considered statistically significant. Overlapping DEGs were detected with the online tool Draw Venn Diagram (http://bioinformatics.psb.ugent.be/webtools/Venn/).

### Functional and pathway enrichment analyses

GO enrichment and KEGG pathway analyses were performed for the identification of DEGs. R packages (clusterProfile, ggplot2 and GOplot) were used ^40^. ClusterProfile package was used in analyzing the DEGs. Ggplot2 (https://ggplot2.tidyverse.org) and GOplot packages were used for the visualization of the results.

### Construction of a PPI network

The common DEGs were analyzed by the online tool STRING (https:// string-db.org) for the construction of a PPI network. The cut-off standard was set as a combined score > 0.4^41^. Then, the results were visualized with CytoScape software. MCODE V1.5.1, which is a plug-in of CytoScape, was used in identifying significant modules (MCODE score ≥ 4)^42^. Moreover, the hub genes were selected using CytoHubba, which is another plug-in of CytoScape, according to the number of associations with other genes in the PPI network^43^. Seven common algorithms (MCC, MNC, Degree, Closeness, Radiality, Stress, and EPC) were used in evaluating and selecting hub genes.

### Prediction of TFs

TRRUST, a database for the prediction of transcriptional regulatory networks, was used in predicting TFs that regulate hub genes, and an adjusted P value of < 0.05 was considered significant^44^.

### Statistical analysis

RStudio (https://www.R-project.org) was used in statistical analysis. GSE12021 and GSE100927 were used in validating the expression levels of the identified hub genes and TFs. Wilcoxon rank sum test was used as the statistical method when the data was not normally distributed, and T- test was used as the statistical method when the dada was normally distributed. P value < 0.05 was considered significant.

## Supplementary Information

Supplementary Information 1.

Supplementary Information 2.

## Data Availability

The datasets generated and/or analyzed during the current study are available in the GEO repository. It is a public free repository database, which stores a large number of gene functions and expressions. The working links are as following, GSE55235(https://www.ncbi.nlm.nih.gov/geo/query/acc.cgi?acc=GSE55235), GSE55457(https://www.ncbi.nlm.nih.gov/geo/query/acc.cgi?acc=GSE55457), GSE12021(https://www.ncbi.nlm.nih.gov/geo/query/acc.cgi?acc=GSE12021), GSE28829(https://www.ncbi.nlm.nih.gov/geo/query/acc.cgi?acc = GSE28829), and GSE100927(https://www.ncbi.nlm.nih.gov/geo/query/acc.cgi?acc = GSE100927).
