# Supplementary figures and images for: Identification of hub genes and transcription factors in patients with rheumatoid arthritis complicated with atherosclerosis
Source: Sci Rep. 2022 Mar 18;12:4677. doi: 10.1038/s41598-022-08274-1 (PMC8933589; doi:10.1038/s41598-022-08274-1)

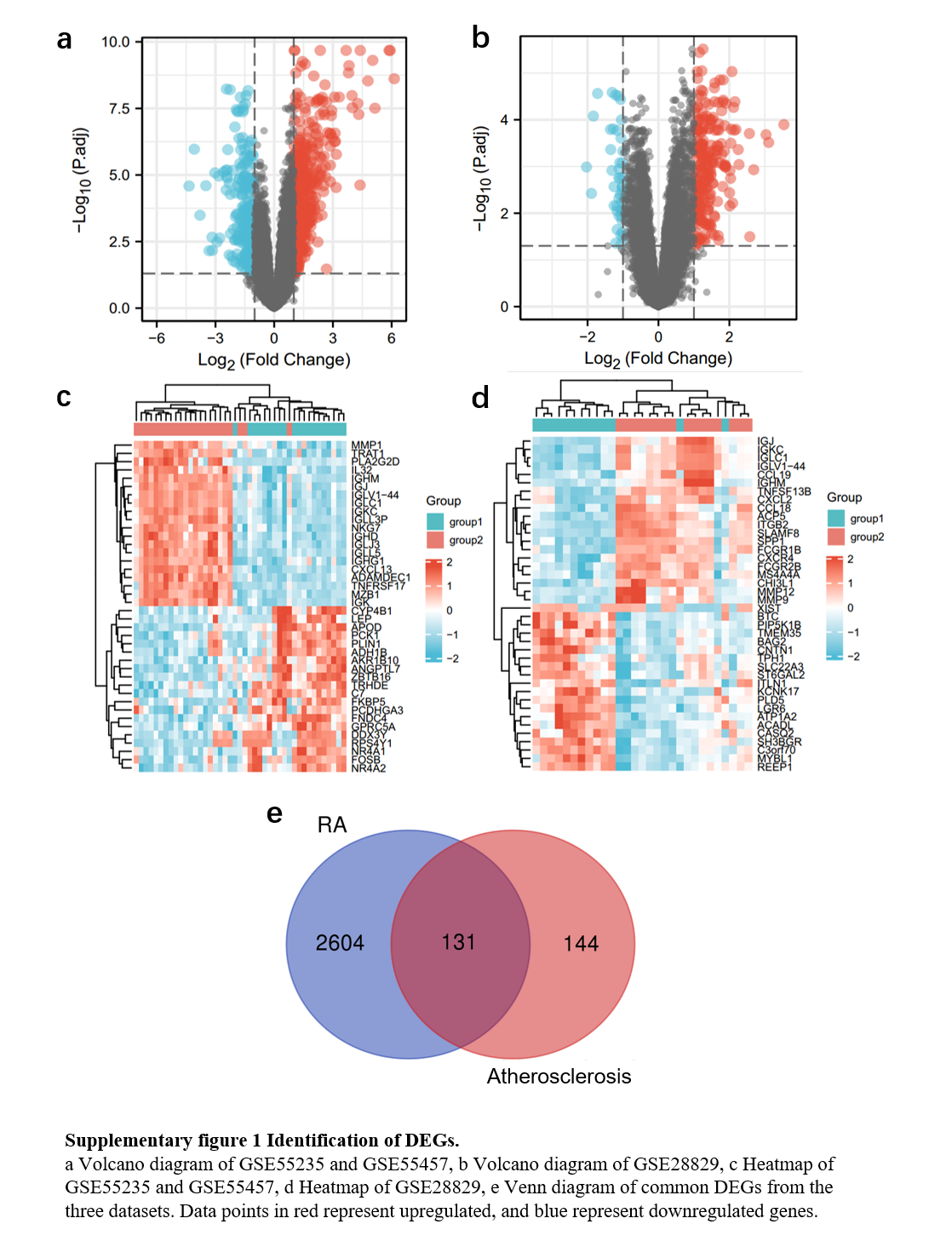

Supplement: Supplementary file 1 — Supplementary Information 1. [file 41598_2022_8274_MOESM1_ESM.png]

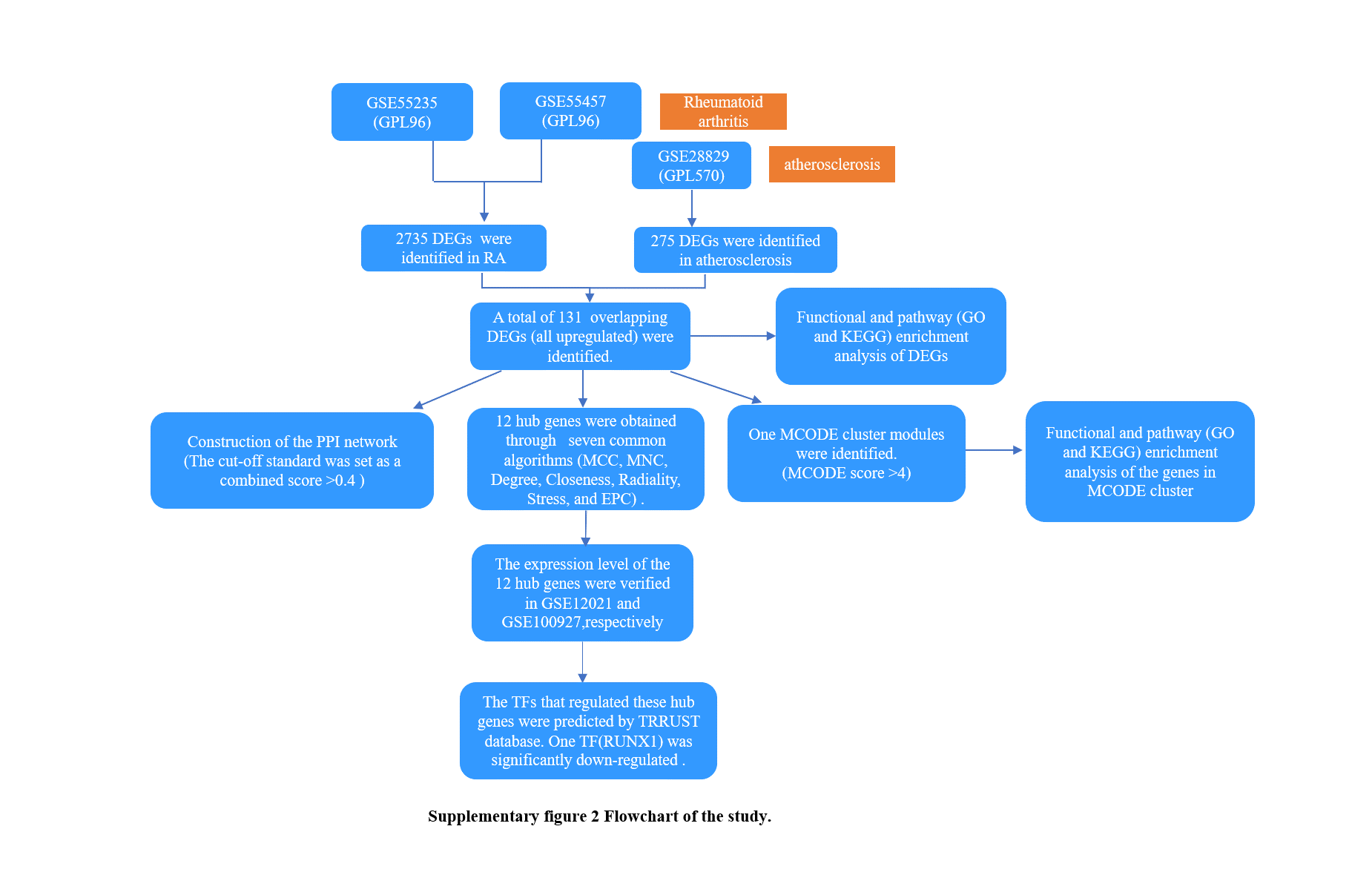

Supplement: Supplementary file 2 — Supplementary Information 2. [file 41598_2022_8274_MOESM2_ESM.png]
